# Indian older adults and the digit span A preliminary report

**DOI:** 10.1590/1980-57642018dn13-010013

**Published:** 2019

**Authors:** Ravikesh Tripathi, Keshav Kumar, Srikala Bharath, Marimuthu P, Vikram Singh Rawat, Mathew Varghese

**Affiliations:** 1 Gujarat Forensic Sciences University Ringgold standard institution – Clinical Psychology IBS, GFSU, Gandhinagar, Ganhinagar 382007, India. Department of Clinical Psychology.; 2 Clinical Psychology; 3 Psychiatry and Biostatistics, National Institute of Mental Health and Neuro Sciences. Bangalore. India.; 4 Biostatistics, National Institute of Mental Health and Neuro Sciences. Bangalore. India. National Institute of Mental Health and Neuro Sciences Department of Biostatistics Ringgold standard institution – Biostatistics, Bangalore, Karnataka, India.; 5 All India Institute of Medical Sciences – Rishikesh Ringgold standard institution – Psychiatry, Rishikesh, Uttarakhand, India.; 6 Psychiatry and Biostatistics, National Institute of Mental Health and Neuro Sciences. Bangalore. India.

**Keywords:** digit span, working memory, span task, older adults, digito span, memória de trabalho, tarefa span, idosos

## Abstract

The digit span test is widely used to assess attention and working memory. It is a portable, relatively culture-free and frequently used test. However, the cultural validity of this test, particularly in the Indian older population, is not well established.

**Objective:**

This study explores the usefulness of the digit span test for Indian older adults with different levels of education.

**Methods:**

Two hundred and fifty-eight community-dwelling healthy normal older adults formed the sample of this study. All study participants were screened using a semi-structured interview schedule, the modified MINI Screen, the Indian version of the Mini-Mental State Examination, a measure of activities of daily living and the digit span test administered verbally.

**Results:**

The results indicated that participants with higher educational level performed significantly better than low-educated participants on the digit span test. Participants with low education often struggled with the digit span test and resorted to guessing the digits.

**Conclusion:**

Our study clearly demonstrates that the digit span test can be useful for educated participants. However, its usefulness and ecological validity is questionable for those with low education and low literacy, warranting future research.

## RESUMO.

O teste de span de dígitos é amplamente utilizado para avaliar a atenção e a memória de trabalho. É um teste portátil, relativamente livre de cultura e frequentemente usado. No entanto, a validade cultural deste teste, particularmente na população idosa indiana, não está bem estabelecida.

**Objetivo:**

Este estudo explora a utilidade do teste de amplitude de dígitos para idosos indianos com diferentes níveis de educação.

**Métodos:**

Duzentos e cinquenta e oito idosos saudáveis ​​normais residentes na comunidade formaram a amostra deste estudo. Todos os participantes da pesquisa foram selecionados usando uma entrevista semiestruturado, MINI Screen modificado, versão indiana do Mini Mental State Examination, uma medida de atividade da vida diária e teste do span de dígitos administrados verbalmente.

**Resultados:**

Os resultados indicaram que os participantes com alto nível de escolaridade tiveram desempenho significativamente melhor do que os participantes com baixo nível de escolaridade no teste de amplitude de dígitos. Participantes com baixa escolaridade muitas vezes enfrentaram difficuldades com o teste de amplitude de dígitos e recorreram a adivinhar os dígitos.

**Conclusão:**

Nosso estudo demonstra claramente que o teste de amplitude de dígitos pode ser útil para participantes instruídos. No entanto, sua utilidade e validade ecológica são questionáveis ​​para aqueles com baixa escolaridade e letramento, requerendo pesquisas futuras.

The digit span test is widely used to assess attention-concentration and working memory.^1^^-^ 3 It is quick, portable and easy to use, often forming an integral part of neuropsychological examinations and the mental state examination.^1^ The Digit span test comprises both digit forward and digit backward conditions. The test involves reading out a series of strings of digits to the participants who are required to repeat them in the same or reverse order of presentation. Performance on the Digit span test requires auditory attention as well as short-term verbal memory.^3^ Better performance on the Digit span test hinges more on the integrity of the left hemisphere than on either right-hemisphere or diffuse damage.^2^

It has been demonstrated that culture and demographic variables can affect performance on the Digit span test.^1^^-^ 5 Indian older adults differ from western counterparts in terms of culture, literacy and exposure to psychological testing.^4^^,^ 5 There is a paucity of research on the usefulness of the digit span, especially amongst elderly. However, available research on other cognitive tests indicates that higher educational level is associated with better performance on several cognitive tests.^4^^-^ 7 It has been observed that Indian older participants are less familiar with psychological testing, feel anxious and may perform poorly on psychological tests.^4^^,^ 8 There are several empirical studies from the Indian subcontinent indicating that older participants perform poorly on several cognitive tests.^4^^,^ 5 ^,^ 9 ^,^ 10

The Digit span test is widely used in Indian settings by researchers as well as clinicians to assess attention and working memory. There is a growing body of evidence suggesting that older healthy participants from developing countries perform poorly on neuropsychological measures 4 ^,^ 8 ^,^ 10 and, therefore, examining cultural validity for neuropsychological measures has been recommended by some researchers. 5 ^,^ 6 ^,^ 11 To the best of our knowledge, there is no study with regard to the cultural validity and usefulness of digit span tests for Indian older participants. In this study, we explored the usefulness of the digit span test for Indian older adults with heterogeneous educational background.

## METHODS

### Participants

Two hundred and fifty-eight community-dwelling healthy normal older adults formed the sample of this study. All participants were volunteers who provided written consent to participate in the present study. No incentives were offered for participation. The study was approved by the institute´s ethics committee.

### Measures

Socio-demographic data sheet: A basic datasheet was used to collect socio-demographic details such as age, sex, date of birth, language, income, address, urban/rural, presence or absence of known physical, neurological or psychiatric illness, past psychiatric/neurological consultation, medication history, and presence of any physical health problem.

**Hindi Mental Status Examination (HMSE):** The HMSE is a global cognitive screen, adapted for Indian older adults.^12^ HMSE is a modified version of Mini-Mental State Examination validated for the Indian population. In this study, the HMSE was used to screen participants with cognitive impairment (≤19 for 0 to 5 years of schooling; <25 for 6 years or higher).

**Everyday Abilities Scale for India (EASI):** This is a 12-item brief measure of activities of daily living with norms, and is appropriate for use in evaluating dementia (together with other tests) in elderly people in India.^13^ We used this scale to screen participants with difficulties in activities of daily living. Higher score on the EASI indicates greater overall disability.

**Digit span:** This is a test of attention/short-term memory involving strings/series of digits (numbers) of varying length.^2^ It consists of 6 items each for forward and reverse assessments, respectively. Further, each item has two trials.

All research participants were assessed individually by a trained clinical neuropsychologist (RT). All participants completed a semi-structured interview schedule, the Indian version of the Mini-Mental State Examination (HMSE), a measure of activity of daily living (EASI) and the modified MINI Screen, followed by the Digit span test. In our study, we excluded participants with known history of major psychiatric or neurological illness.

The Statistical Package for the Social Sciences (SPSS 16.0) was used to analyse the data obtained. Descriptive statistics by years of education, gender, age and occupation were reported. Stepwise multiple regression analysis was used to examine the contribution of age, gender and education on digit span.

## RESULTS

Two hundred and fifty-eight community-dwelling healthy older adults aged 50 to 80 years participated in the present study. The mean age and education of the sample was 62 years (SD=6.44) and 12 years (SD=5.25), respectively. The demographic data of the participants are depicted in Table 1. Mean scores on the digit span (forward and backward) are also presented in Table 1. The results show that the older participants recalled 5.60±1.22 digits forward and 4.00±1.40 digits backward (Table 2). Illiterate and low-educated participants performed significantly poorer on the digit span test.

**Table 1:** Demographic data of the participants, with means and standard deviations.

		N	Forward			Backward	
			M	SD		M	SD
Age group	50-59	112	5.51	1.21		4.06	1.50
	60-69	112	5.60	1.24		4.09	1.47
	70-80	34	5.57	1.19		4.18	1.04
Gender	Male	189	5.76	1.24		4.27	1.34
	Female	69	5.11	1.03		3.60	1.56
Education	Illiterate	19	4.00	1.00		1.50	1.50
	1-5 Years	14	4.35	1.00		2.64	1.33
	6-8 Years	17	5.35	1.32		3.82	1.01
	9-12 Years	59	5.23	1.20		3.70	1.02
	13-15 Years	83	6.00	1.00		4.65	1.00
	≥16 Years	66	6.20	1.04		4.90	1.18
Occupation	Professional	110	6.17	1.00		4.70	1.10
	Clerical	69	5.60	1.20		4.30	1.15
	Household	79	4.70	1.00		3.10	1.70
Total		258	5.59	1.22		4.09	1.43

**Table 2:** Global cognitive score, everyday abilities and digit span in older adults: means and standard deviations (N=258).

	Mean	SD
Hindi Mental Status Examination	29.79	2.00
Everyday Abilities Scale for India	0.00	0.00
Digit span (Forward)	5.60	1.22
Digit span (Backward)	4.00	1.40

Stepwise regression analysis was performed to examine the contribution of age, education, gender and occupation on digit span test scores. Table 3 shows the contribution of demographic factors on digit span test scores. Education was found to be a strong contributor, having a significant effect on the digit forward and backward, followed by occupation. Education accounted for 32% and 42% of the variance for digit forward and backward, respectively. Occupation was significantly associated with better performance on the digit span forward, accounting for 3% of the variance. In this study, the contributions of age and gender were not statistically significant.

**Table 3:** Contribution of age, education and gender on Digit span.

Dependent variables	Independent variables	B	T	P	R^2^	Proportion of attribution
Digit span (Forward)	Education	0.40	5.85	0.00	0.35	0.32
	Occupation	-0.24	-3.53	0.00		0.03
Digit span (Backward)	Education	0.65	13.66	0.00	0.42	0.42

## DISCUSSION

The Digit span test is widely used to assess attention and working memory and is considered an integral part of neuropsychological evaluation. To our knowledge, there is a lack of information regarding the usefulness of the digit span test for Indian older adults. The main purpose of this study was to examine the utility of the digit span test for older Indian adults.

Our findings suggest that education was positively associated with better performance on the digit span test. Higher educated participants performed significantly better than low-educated participants on the digit span (forward and backward). Moreover, in our study, education emerged as the strongest determinant of digit span performance and this finding is consistent with previous studies.^1^^,^ 3 ^,^ 5 Interestingly, in the current study, previous occupation was meaningfully associated with the digit forward. However, it did not emerge as a significant predictor for digit backward. This finding should come as no surprise, as better education may provide greater occupational opportunity/achievement which, in turn, may facilitate better cognitive reserve. Consistent with our findings, Ryan, Lopez and Paolo (1996) found that occupation was meaningfully associated with digit span performance.

In this study, we found that participants with lower levels of education, as well as illiterate individuals, performed poorly on the test, especially the backward span. The majority of the low-educated participants were able to repeat a maximum of 4 digits forward adequately. However, under the backward condition, low-educated groups performed worse than their higher-educated counterparts. Most of the illiterate participants refused to participate or cooperate on the digit span backward task. This could have been due to lack of exposure to mental operations in their everyday activities. The refusal to participate calls for further exploration. The Digit span forward is a somewhat easier task and requires attention and concentration. Consequently, the majority of the illiterate and low-educated participants performed well on this task. On the other hand, the digit backward condition, in which a sequence of digits has to be repeated in the reverse order, requires more effortful processing and relies on central executive resources. It is known that education improves executive functioning^14^ and, therefore, educated participants perform better on this task.

In our study, we observed that several participants with low education were able to improve with repeated trials. This might be due to unfamiliarity with the test, as well as the procedure required for this task. It was observed that test administration provided an opportunity for many illiterate and low-educated participants to learn and experiment with these mental operations for the first time in their lives. Another striking observation was that several participants were unable to pass initial or easy items, but could pass higher items if the trial was not discontinued. This is suggestive of “intra-test scatter” in these participants, where this might be due to anxiety consequent to unfamiliarity with such tasks.

The presence of intra-test scatter observed in our study, may warrant modification in the standardization procedure for individuals with no formal education or low levels of education. However, in this study we were not able to modify the standardized procedures; future studies are required to examine such procedural changes and their impact on performance. We would like to highlight the fact that poor performance by the participants on the digit span test may not necessarily indicate disturbances in brain functions. In view of their ability to improve their performance with practice, a practice trial may be warranted for participants with lower levels of education or illiterate individuals. Caution needs to be exercised while using and interpreting the digit span with low-educated participants. Education certainly helps to learn, develop strategies and attitude that could result in a better cognitive reserve. However, several other factors such as anxiety, task familiarity and the cultural value system in the context of psychological testing could also result in poor performance on digit span tests.

Based on our findings, we suggest that the digit span test may not be an ecologically valid test for low-educated participants, including illiterate subjects. The majority of the low-educated healthy participants performed poorly on the digit span test, particularly on the digit backward task. We would like to recommend further research on the development of longitudinal robust normative data for Indian older participants, including illiterate and literate participants.

This study had several limitations. It involved older adults and, therefore, the results of the present study cannot be generalized to younger participants. In our study sample, the number of female participants with low literacy was relatively small. Future studies are needed to confirm the findings.

In conclusion, in this study, we examined the usefulness of the digit span test involving a cohort with heterogeneous education. We found that Indian participants with low education performed poorly, despite no apparent brain dysfunction, and often resorted to guessing the digits on items containing larger strings of digits, particularly for the backward condition. Our study clearly demonstrates that the digit span test can be a useful test for educated participants. However, when testing individuals with lower literacy levels, caution needs to be exercised in terms of assessment, as well as interpretation, of the test. Several practice trials may be necessary, as these individuals seem to learn during the test and possibly improve their performance.
